# Sex Differences in the Association between Level of Childhood Interleukin-6 and Insulin Resistance in Adolescence

**DOI:** 10.1155/2012/859186

**Published:** 2012-01-05

**Authors:** Anna Bugge, Bianca El-Naaman, Robert G. McMurray, Karsten Froberg, Claus Henrik Nielsen, Klaus Müller, Lars Bo Andersen

**Affiliations:** ^1^Centre for Research in Childhood Health, Institute of Sports Science and Clinical Biomechanics, University of Southern Denmark, Campusvej 55, 5230 Odense M, Denmark; ^2^Department of Exercise and Sport Science, University of North Carolina, Fetzer Gym, South Road, Chapel Hill, NC 27599-8700, USA; ^3^Department of Infectious Diseases and Rheumatology, Institute for Inflammation Research, Rigshospitalet, Blegdamsvej 9, 2100 Copenhagen Ø, Denmark; ^4^Pediatric Clinic II, Rigshospitalet, Blegdamsvej 9, 2100 Copenhagen Ø, Denmark; ^5^Department of Sports Medicine, Norwegian School of Sport Sciences, Sognsveien 220, 0806 Oslo, Norway

## Abstract

The purpose of this study was to determine whether levels of interleukin-6 (IL-6) in childhood are related to insulin resistance in adolescence. Further, to explore how fatness and cardiorespiratory fitness (VO_2peak_) moderate this relationship. *Methods*. 292 nine-year-old children (*n* = 292) were followed for 4 years. Anthropometrics and VO_2peak_ were measured. Fasting blood samples were analyzed for IL-6, insulin, and glucose. Homeostasis model assessment (HOMA-IR) was used as a measure of insulin resistance. *Results*. For girls but not boys, levels of IL-6 at age 9 yrs correlated with HOMA-IR at age 13 yrs: *r* = 0.223, *P* = 0.008. Girls with IL-6 levels within the highest quartile at age 9 yrs had an odds ratio of 3.68 (CI = 1.58–8.57) being in the highest quartile of HOMA-IR four years later. *Conclusion*. In this cohort, IL-6 levels in childhood were related to insulin resistance in adolescence, but only for girls.

## 1. Background

It is well established that increased levels of fatness are associated with low-grade inflammation in adults [1, 2], as well as in children [3]. Low-grade inflammation has been proposed as a mechanism linking obesity with systemic complications such as insulin resistance and type 2 diabetes [4, 5]. Among the inflammatory markers, IL-6 has emerged as one of the potential mediators between obesity and insulin resistance/diabetes [6–8]. However, reports have suggested both pro- and anti-inflammatory effects of IL-6 [9, 10]. IL-6 is a cytokine produced in various tissues including adipose tissue, muscle tissue, and immune cells. Resting, circulating levels of IL-6 have been found positively related to fatness [7, 11] and negatively related to the level of fitness and physical activity [12].

The cross-sectional relationship between IL-6 and insulin resistance in youth has been investigated, but no relationship has been found [13–16]. One longitudinal study, investigated the effect of a weight loss program on inflammatory markers in obese children [17]. They found a weak but significant correlation between change scores of IL-6 and HOMA-IR over one year. To our knowledge, no studies have investigated the longitudinal relation between IL-6 and HOMA over the course of childhood and early adolescence in a normal pediatric population. Therefore, the aim of the present study was to evaluate associations between IL-6 and HOMA-IR in a longitudinal design, following children through early adolescence. Furthermore, we aimed at exploring whether fatness or VO_2peak_ moderate these relationships. We hypothesized that in a normal pediatric population (1) a high level of IL-6 in childhood is, not was related to an elevated HOMA-IR in adolescence and (2) a high level of IL-6 in childhood tracks into adolescence.

## 2. Methods

### 2.1. Participants

All children in kindergarten classes (6-7 years of age) from 2 suburbs of Copenhagen, Denmark, were invited to participate in a 3-year, controlled intervention study of physical activity and health: the Copenhagen School Child Intervention Study. Data were collected at baseline, following the intervention, and 4 years after the intervention. The study has been described in details elsewhere [18, 19]. Data used in this paper is from the postintervention measurement in 2003/2004 (age 9 yrs) and the follow-up measurement in 2008 (age 13 yrs). Complete data on IL-6 from both assessments were available for 292 subjects. Since no differences between IL-6 levels in the intervention and control group were found, data from the two groups were pooled for all analyses.

### 2.2. Ethics

The ethical committee of Copenhagen approved the study. Written informed consent was obtained from the parents/guardians.

### 2.3. Measurements

Tests were performed at all 18 schools in the two communities. Fitness test was performed in a mobile unit, and all other tests were performed in a gym or a classroom between 8:00 AM and 02:00 PM. Fasting blood samples were collected before 10 AM. Body mass was measured to the nearest 0.1 kg (Seca 882, Brooklyn, NY). Body height was measured to the nearest 1 mm in bare feet (Harpenden stadiometer, West Sussex, UK). Body mass index (BMI) was calculated as weight multiplied by height squared. Skinfolds (biceps, triceps, subscapular and suprailiac) were measured to the nearest mm with caliper (Harpenden, West Sussex, UK) according to criteria presented by De Lorenzo and coworkers [20]. The mean of 3 measurements was used for subsequent analysis. The sum of four skinfolds (S4SF) was calculated and used as an estimate of body fatness [21]. Waist circumference was measured at the end of a normal expiration, midway between the lower rib margin and the anterior iliac crest. Sexual maturation was assessed by self-report using a scale of pictures of breast and genital development for girls and boys, respectively [22]. 5 boys and one girl were unwilling to have their sexual maturation assessed.

Blood samples were collected in the morning after a minimum of 8 hours verified fasting. Children did not have their blood drawn if they suffered from any infectious disease or had fever. Samples were immediately analyzed for glucose (Hemocue, Sweden). The rest of the samples were centrifuged, and the plasma was initially frozen at −20°C and then stored at −80°C the same day. Insulin was analyzed using an enzyme-linked immunosorbent assay (DAKO Insulin, Code no. K6219; DAKO, Glostrup, Denmark). Homeostasis model assessment (HOMA-IR) was used as an estimate of insulin resistance [23]: ([glucose (mmol·L^−1^) × insulin (mU·L^−1^)]/22.5). IL-6 was analyzed with high-sensitivity immunoassays (Quantikine High-Sensitivity ELISA) with a detection limit of 0.5 pg·mL^−1^ (R&D, Minneapolis, MN). Cardiorespiratory fitness (VO_2peak_) was measured during a progressive treadmill running test until exhaustion. VO_2peak_ was measured using an AMIS 2001 Cardiopulmonary Function Test System (Innovision, DK 5260 Odense) at age 9 yrs and using the COSMED K4b^2^ portable metabolic system (COSMED, Rome, IT) at age 13 yrs. Both systems have been found to give reliable measures of VO_2_ uptake, when validated against the Douglas bag method [24, 25]. For a test to be valid, at least one of three objective physiological criteria should be fulfilled; heart rate higher than 200 beats/min, respiratory exchange ratio equal to or higher than 1.00, or a plateau of VO_2_ defined as an increase of less than 2.1 mL*·*min^−1^·kg^−1^ [26]. Moreover, the test leader should subjectively consider the child exhausted. If a child ran to exhaustion, but did not attain a valid measurement only because of equipment failure, we estimated VO_2peak_ from running time to exhaustion and sex in a regression equation using all the valid measurements, as earlier reported [19]. Approximately 10% of children had their VO_2peak_ calculated.

### 2.4. Data Analysis

Means and standard deviations for all variables were computed by sex for descriptive purposes. BMI, waist circumference, S4SF, HOMA-IR, and IL-6 were positively skewed and therefore transformed (natural log) for the analyses. Independent samples *t*-tests were used to test for differences between sexes.

Pearson correlations were used for longitudinal bivariate associations between IL-6 at age 9 yrs and HOMA-IR at age 13 yrs, adjusting for HOMA-IR at age 9 yrs. Then, in a stepwise fashion the correlation were adjusted for (1) S4SF (2) fitness, and (3) both S4SF and fitness at age 13 yrs. Subsequently, children were divided into groups based on their maturation (Tanner stages 1–3 and 4-5), and the correlations were performed stratified for this variable. To further explore the data, logistic regression was performed. The children were divided into sex-specific quartiles of HOMA-IR and IL-6 for the logistic regression. Odds ratios were calculated for being in the upper quartiles of IL-6 and HOMA-IR at age 13 yrs, based on being in the upper quartile of IL-6 at age 9 yrs, compared to the three lower quartiles. The analyses of HOMA were adjusted for HOMA at age 9 yrs to account for any differences already present at baseline. Finally, cross-sectional correlations between IL-6 and HOMA-IR both at age 9 yrs and at age 13 yrs were assessed and adjusted for (1) S4SF, (2) fitness, and (3) S4SF and fitness. All analyses were performed using the statistical package for the social sciences version 15 (SPSS, Chicago, IL).

## 3. Results

The general characteristics of the samples at the age of 9 and 13 yrs are presented by sex in Table 1. Generally, boys were slightly older and taller than girls and had a greater waist circumference (*P* < 0.05). Boys had a greater aerobic fitness and lower S4SF (both *P* < 0.001), as well as lower HOMA-IR (*P* < 0.02), compared to girls. At ages 9 and 13 yrs, the girls were on average ~0.4 Tanner stages ahead of the boys (*P* < 0.001).

Relationships between circulating IL-6 levels at age 9 yrs and IL-6 and HOMA-IR at age 13 yrs are presented in Table 2. A significant correlation was found between levels of IL-6 at age 9 yrs and at age 13 yrs for the girls, but not for the boys. Furthermore, the girls also displayed correlations between IL-6 levels at age 9 yrs and HOMA-IR four years later. These correlations were weakened after adjusting for fatness, and fitness and fatness combined at age 13 yrs.  Table 3 shows these correlations stratified by Tanner stages. Only the most mature girls (Tanner stages 4-5) displayed a significant correlation between IL-6 levels at age 9 yrs and HOMA-IR at age 13 yrs.

Logistic regression was performed to assess the risk of ending up in the highest quartile of HOMA-IR at age 13 yrs after having had systemic IL-6 levels within the upper quartile at age 9 yrs. The results are presented in Table 4. Girls with IL-6 levels within the upper quartile at age 9 yrs had an odds ratio of 2.55 for having IL-6 levels within the upper quartile at age 13 yrs and an odds ratio of 3.86 for being in the upper quartile of HOMA-IR, compared with the rest of the girls. There were no other significant increased risks (*P* > 0.05).

There were no significant cross-sectional correlations between IL-6 levels and HOMA-IR at age 9 yrs or at age 13 yrs, irrespective of adjustment for fatness and fitness. HOMA age 9 yrs was correlated with HOMA age 13 yrs for girls (*r* = 0.242, *P* = 0.004), but not for boys (*r* = 0.143, *P* = 0.083).

## 4. Discussion

To our knowledge this is the first study to examine the longitudinal relation between IL-6 and HOMA-IR in a normal pediatric population. A correlation was observed between circulating IL-6 levels at age 9 yrs and HOMA-IR at age 13 yrs for girls, but not for boys. In support of a longitudinal relationship between IL-6 and HOMA-IR, Roth and colleagues found a weak but significant correlation between change scores of IL-6 and HOMA-IR over one year in an intervention study on obese children [17]. This result, together with our finding in girls, could suggest a role of IL-6 in the longitudinal development of insulin resistance. However, we did not find any correlations between IL-6 and HOMA-IR in the cross-sectional analyses, in accordance with findings of other cross-sectional studies on IL-6 and insulin level or HOMA-IR in youth [13–15, 27].

The specific role of IL-6 in the pathogenesis of insulin resistance is still controversial. It has been shown that IL-6 suppresses the production of TNF-*α*, a cytokine involved in the pathogenesis of insulin resistance and CVD [10]. Concomitantly, TNF-*α* causes IL-6 production and release of IL-6 to the circulation [9]. Therefore, hypothetically it is possible that TNF-*α* actually induces impaired glucose metabolism, while high systemic levels of IL-6 reflect a high local production of TNF-*α* and are not directly involved in the pathogenesis of insulin resistance [9]. Unfortunately, we were not able to include other measures of low-grade inflammation, for example, TNF-*α*, TNF-modifying mediators, or CRP in this study, which makes it impossible for us to make any interpretations regarding the mechanisms by which low-grade inflammation influence the development of insulin resistance. Our findings should therefore just be interpreted as descriptive.

Body fat is related to HOMA-score [28], as well as to systemic IL-6 levels in some studies of pediatric populations [16, 29–31], but not all [14, 17, 27, 32]. Fatness could therefore potentially influence the relation between IL-6 and HOMA-IR. Adjusting for S4SF at age 13 yrs weakened the correlation found in girls between IL-6 at age 9 yrs and HOMA-IR at age 13 yrs in this study, but systemic IL-6 levels remained as an independent predictor of high HOMA-IR at age 13 yrs. A study by Tam and colleagues found no significant differences in IL-6 level between normal weight and overweight/obese groups at age 8 yrs, but when followed up at age 15 yrs, overweight/obese girls had significantly higher levels of IL-6, compared to normal-weight girls. No differences were seen for boys [33]. This study supports our finding of a sex difference in relation to IL-6, which has also been found in studies on adult populations [34, 35]. Furthermore, their results, together with our results, suggest that in girls elevated IL-6 production develops over a prolonged period of time and is, possibly, influenced by sexual maturation or estrogens. The girls in our study were on average 0.4 tanner stages ahead of the boys at the same age (Table 1), which may account for some of the sex differences we found in the relations between IL-6 and HOMA-IR. When the girls in our study were split into groups of maturation stages (Tanner stages 1–3 and Tanner stages 4-5), only the mature girls displayed a significant correlation between IL-6 and HOMA-IR (Table 3). However, this difference in significant findings between sexual maturation groups was probably caused by a lack of power due to the small sample size in the less mature group. Actually, the correlation coefficient in the less mature group was higher compared to the more mature.

In the present study, adjustment for VO_2peak_ did not alter the correlations between IL-6 and HOMA-IR. Likewise, Rubin et al. did not find any effect of adjusting for maximal aerobic power in the association between HOMA-IR and IL-6 [13]. Other studies have investigated the relationship between IL-6 and fitness in youth and did not find any correlation [15, 16].

The main strength of the present study is the longitudinal design with a four-year period between the two measurements. To our knowledge, no other researchers have followed children for this length of time. One limitation of this study is the low number of overweight and obese individuals in the study population and low mean values for both HOMA-IR and IL-6 levels (Table 1). However, we have earlier reported that in this cohort ~14% of the children had an adverse CVD risk profile at age 9 yrs [36]. Also, we did not include other inflammatory markers, for example, TNF-*α* and TNF-modifying mediators in this study, thus we cannot exclude that some confounders related to both IL-6 and insulin resistance are affecting the associations we investigated. Therefore, no conclusions regarding the mechanisms by which low-grade inflammation affect insulin resistance can be drawn. Future studies could therefore focus on other inflammatory markers more closely related to the pathogenic processes in youth, for example, CRP and TNF-*α*. Furthermore, a more diverse population including a higher number of overweight and obese children and more children with adverse health outcomes could strengthen the scientific impact of the results. Also the genetic aspects in the relation between low-grade inflammation and the pathogenesis of insulin resistance should be investigated in future studies. Finally, we did not control for exercise immediately prior to blood sampling. However, blood sampling was done early in the morning, and it was not likely that any of the children had performed prolonged exercise prior to sampling. Furthermore, although we excluded children with any signs of infections or fever, undetected subclinical infection could have caused an increase in IL-6 levels in some children. We did, however, try to run all analyses omitting outliers and that did not change our results substantially.

In conclusion, the results of this study show that IL-6 levels in childhood track into adolescence, especially for girls. Further, IL-6 levels in childhood are related to HOMA-IR four years later for girls, but not for boys. These relationships remained significant after adjusting for fatness and fitness. We did not find any cross-sectional association between IL-6 and HOMA-IR at either age 9 or age 13 yrs, suggesting that in these age groups the level of IL-6 is not directly related to HOMA-IR.

## Figures and Tables

**Table 1:** Characteristics of participants by sex and age: mean and standard deviation.

Variables	Boys		Girls	
	9 years	13 years	9 years	13 years
Age (years)	9.6 (0.4)	13.4 (0.3)	9.5 (0.3)	13.3 (0.3)
Height (cm)*	140.6 (5.7)	164.9 (8.0)	138.8 (6.8)	162.2 (6.8)
Weight (kg)	33.7 (6.0)	51.7 (10.0)	32.7 (6.1)	50.5 (8.5)
BMI (kg·m^−2^)	17.0 (2.2)	18.9 (2.7)	16.9 (2.2)	19.2 (2.6)
Normal weight/overweight/obese (%)	88.1/9.9/2.0	88.7/9.3/2.0	87.2/12.1/0.7	90.7/9.3/0.0
Waist circumference (cm)*	62.1 (6.3)	68.3 (7.0)	60.6 (6.2)	65.7 (5.4)
Sum of four skinfolds (mm)*	27.5 (12.5)	31.2 (17.8)	33.8 (15.8)	37.4 (15.6)
Tanner stages (1–5)	1.1 (0.2)	3.4 (0.8)	1.4 (0.6)	3.8 (0.8)
Aerobic fitness (mL·kg^−1^·min^−1^)*	53.4 (6.8)	53.0 (7.9)	47.6 (5.9)	45.7 (7.2)
IL-6 (pg·mL^−1^)	0.8 (1.0)	0.6 (0.5)	1.1 (1.3)	0.8 (1.4)
HOMA score*	1.2 (0.6)	2.5 (1.4)	1.3 (0.7)	2.9 (1.5)

*P < 0.05, girls versus boys.

**Table 2:** Pearson correlations between IL-6 at age 9 yrs and IL-6 and HOMA-IR at age 13 yrs, (1) unadjusted, (2) adjusted for sum of 4 skinfolds (S4SF), (3) VO_2peak_ (mL/kg), and (4) both S4SF and VO_2peak_ at age 13 yrs.

Risk factors age 13 yrs	IL-6 age 9 yrs			
	Unadjusted	Adjusted for S4SF	Adjusted for VO_2peak_	Adjusted for S4SF & VO_2peak_
IL-6				
Boys	0.130	0.138	0.136	0.133
Girls	0.360*	0.349*	0.402*	0.388*
HOMA-IR^a^				
Boys	0.053	0.065	0.044	0.055
Girls	0.223*	0.199*	0.223*	0.211*

*P value < 0.04. Significant correlations in bold face.

^a^ adjusted for HOMA age 9 yrs.

**Table 3:** Pearson correlations between IL-6 at age 9 yrs and IL-6 and HOMA-IR at age 13 yrs in groups based on sexual maturation and adjusted for HOMA age 9 yrs.

		*n*	*r*
Boys	Tanner stages 1–3	83	0.006
	Tanner stages 4-5	63	0.107
Girls	Tanner stages 1–3	41	0.286^†^
	Tanner stages 4-5	99	0.218*

Significant correlations in bold face. *P value = 0.031. ^†^ *P* value = 0.077, borderline significant.

**Table 4:** Risk of high IL-6 and HOMA-IR level in adolescence based on IL-6 level in childhood.

		95% confidence	
	Odds ratio	Interval	*P* value
IL-6 at age 13			
Boys	0.90	0.38–2.12	0.81
Girls	**2.55**	**1.12–5.80**	**0.03**
HOMA-IR at age 13^a^			
Boys	0.93	0.39–2.22	0.87
Girls	**3.68**	**1.58–8.57**	**0.003**

Odds ratios for being in the highest quartile of CVD risk factors age 13 yrs according to being in the upper quartile of IL-6 at age 9 yrs. Significant odds ratios are presented in bold.

^a^: adjusted for HOMA at age 9 yrs.

## Abbreviations

CVD: Cardiovascular disease
IL-6: Interleukin-6
VO_2peak_: Cardiorespiratory fitness
HOMA-IR: Homeostatic model assessment
CRP: C-reactive protein
TNF-*α*: Tumor necrosis factor-*α*
BMI: Body mass index
S4SF: The sum of four skinfolds.
